# Contamination level and dietary risk assessment of trace elements in *Brassica pekinensis Lour.* from gardening areas under tropical conditions

**DOI:** 10.1016/j.toxrep.2025.102165

**Published:** 2025-11-12

**Authors:** Anaïs M. Kipelo, Masi Jothilakshmi, Dhafer M.M. Al Salah, Georgette N. Ngweme, Emmanuel K. Atibu, Crispin Mulaji, Periyasamy Sivalingam, John W. Poté

**Affiliations:** a National Pedagogic University (UPN), Croisement Route de Matadi et Avenue de la Libération. Quartier Binza/UPN, B.P. 8815, Kinshasa, Democratic Republic of the Congo; b Department of Biotechnology, Bharathiar University, Coimbatore, Tamil Nadu 641046, India; c King Abdulaziz City for Science and Technology, Wellness and Preventative Medicine Institute, Prince Turki the 1st, Riyadh 11442, Saudi Arabia; d School of Public Health, Faculty of Medicine, University of Kinshasa, B.P. 11850, Kinshasa XI Democratic Republic of the Congo; e Faculty of Science, Department of Chemistry, University of Kinshasa, B.P. 190, Kinshasa XI, Democratic Republic of the Congo; f Department of Research and Analytics (DORA), Saveetha Dental College and Hospital, Saveetha Institute of Medical and Technical Sciences (SIMATS), Saveetha University, Chennai, Tamil Nadu 600077, India; g Human Science Research Center (Centre de Recherche en Sciences Humaines (CRESH)), 33, Avenue Comité Urbain, Commune de la Gombe, B.P 3474, Gombe, Kinshasa, Democratic Republic of the Congo; h Faculty of science, F.-A. Forel Department and Institute of Environmental Sciences, University of Geneva, 66 Boulevard Carl-Vogt, Geneva CH – 1205, Switzerland

**Keywords:** Urban agriculture, Irrigation water, *Brassica pekinensis Lour.*, Toxic metals, Dietary intake, Human health risk

## Abstract

This study examined the levels of trace elements in *Brassica pekinensis Lour*. collected from urban gardening sites (Cecomaf and Lutendele) in Kinshasa (Democratic Republic of the Congo) and associated potential human health risks for the consumer. The toxic metal concentrations in leaf samples were analysed using Inductively Coupled Plasma Mass Spectrometry (ICP-MS) and Atomic Absorption Spectrophotometer (AAS). Results showed that Cr, Cu, Cd, Pb, and Hg concentrations in samples from both sites exceeded the FAO/WHO maximum permissible limits, with Cecomaf displaying notably high levels of Cu (115.39 mg·kg⁻¹), Cd (2.95 mg·kg⁻¹), Pb (30.94 mg·kg⁻¹), and Hg (0.058 mg·kg⁻¹). Health risk assessments associated with the consumption of *Brassica pekinensis* L, based on Estimated Daily Intake (EDI), Target Hazard Quotient (THQ), and Hazard Index (HI), indices indicated that EDI values for Cd and Pb exceeded recommended limits in several samples, while THQ values exceeded 1 for most metals except As, suggesting potential non-carcinogenic risks. HI values ranged from 2.98 to 16.11 at Cecomaf and 4.85–12.15 at Lutendele, highlighting a high cumulative risk from individual and multi-metal exposure to consumers. Monte Carlo simulation further confirmed these findings by showing that a significant proportion of the exposed population exceeded the WHO safety thresholds for Cd, Pb, and Cu, thereby reinforcing the robustness of the deterministic risk estimates and illustrating the uncertainty associated with age, sex, and local consumption variability. Spearman correlation analysis revealed strong positive associations between Cr–As, Cr–Pb, and Cd–Hg, suggesting common anthropogenic sources or shared environmental pathways. Results of this study highlight the importance of continuous monitoring of trace elements in leafy vegetables for food safety evaluation, adopting stricter agricultural practices in policy making and management, and public health measures to mitigate long-term exposure risks to urban and peri-urban areas populations.

## Highlights

• Concentration of toxic metals in *Brassica pekinensis Lour* were determined.
• Level of Cr, Cu, Cd, Pb and Hg in plant leaf exceeded FAO/WHO permissible limit.
• THQ values for most metals except As, suggesting potential non-carcinogenic risks.
• Hazard Index highlights great cumulative risk to the plant consumers.
• Further studies for long-term exposure risks to the plant consumers are recommended.

## Graphical Abstract

**Figure:**
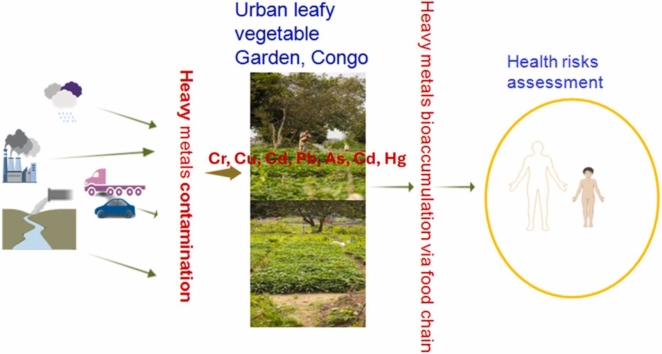

## 1 Introduction

Leafy vegetables, cultivated and consumed worldwide, are a vital part of the human diet [23], rich in trace elements, phytochemicals, vitamins, minerals, and dietary fiber, which provide significant health benefits [15], [54], [66], [9]. On the other hand, it has been demonstrated that leafy vegetables can accumulate high levels of trace elements, particularly in their edible parts [60]. The accumulation of trace elements in plants depends on various factors, such as soil properties, metal properties, plant type, growth stage, climate, and environmental conditions [19], [24].

Although trace elements are naturally occurring elements, they can accumulate over time in plants, especially leafy vegetables, when grown in contaminated soils [3]. The main sources of heavy metal accumulation in plants include industrial activities, mining, smelting and the use of fertilizers and pesticides in agricultural practices [5], [47]. Furthermore, landfill leachate and vehicle emissions near roads, and the natural geological background can contribute to soil, water and vegetable contamination with trace elements [17], [56], [7]. Intriguingly, studies have demonstrated that plant roots and foliage can serve as the primary uptake pathways for trace elements in vegetables [18].

Previous studies reported soil pH could influence the heavy metal transfer to plants [67]. It is still common in many developing countries to use contaminated water for agriculture, which leads to the accumulation of contaminants in the soil [1], [30], [40], [49]. The direct contact with contaminated water and soil subsequently leads to the accumulation of trace elements in leafy vegetables [18], [58], posing high health risks to humans. Studies have shown the consumption of fresh fruits and vegetables can be a primary pathway to human exposure to trace elements via the food chain [42], [53]. For instance, previous studies documented that leafy vegetables can accumulate high levels of trace elements [12], [28]. As a result, numerous studies have investigated heavy metal levels in vegetables and the associated human health risks worldwide [34], [46], [61]

Toxicological studies indicate that regular consumption of vegetables contaminated with trace elements can adversely affect human health, potentially leading to health disorders such as anemia, hypertension, cardiovascular disease, metabolic dysfunction, and neurological issues [55]. In particular, consuming vegetables contaminated with trace elements like Pb, Cu, and Cd has been associated with an increased risk of cancer [27]. Considering the above facts, regulatory authorities mainly the Food and Agriculture Organization (FAO) and World Health Organization (WHO) have recommended dietary threshold limits for trace elements in vegetables. In general, risk assessments for food contaminants often rely on carcinogenic and non-carcinogenic indices [57]. For example, Estimated Daily Intake (EDI), Target Hazard Quotient (THQ), Hazard Index (HI), Carcinogenic Risk Index (CRI), and Total Carcinogenic Risk Index (TCRI), are employed to evaluate the risks of heavy metal exposure from vegetable consumption. For example, a previous study found that both carcinogenic and non-carcinogenic risks exceeded safe thresholds for vegetables grown in the Yamuna River floodplain, India [49]. Kinshasa, the densely populated capital of DRC, has faced intensive anthropogenic pollution in recent years. In this city, the consumption of leafy vegetables like *Brassica pekinensis Lour*, commonly grown in urban farming areas such as Lutendele and Cecomaf, may expose people to trace elements and could potentially negatively affect human health.

The reason for heavy metal contamination in leafy vegetables from these regions is probably due to intensive agricultural practices involving chemical fertilizers, pesticides, and organic amendments like manure. Notably, previous studies have shown high levels of trace elements in the soils, sediments, and irrigation water at the Cecomaf site [39], [47], along with elevated concentrations of persistent organic pollutants, including pesticides [64]. The metals analyzed in this study—Cu, Co, Fe, Ni, Cr, Mn, and Zn—play crucial roles in various physiological and biochemical processes in the human body. However, excessive intake of these elements can lead to toxicity, disrupting protein metabolism and causing numerous health disorders [11]. Furthermore, a previous study demonstrated that, even low-level exposure to toxic metals such as Cd, Hg, Cr, and Pb, through the ingestion of contaminated leafy vegetables, can result in both acute and chronic diseases, including carcinogenic effects [38]. Therefore, it is essential to assess the potential health risks associated with consuming heavy metal-contaminated vegetables. Even though our previous studies indicate heavy metal contamination levels in soils and irrigation water in Kinshasa, the accumulation of trace elements in leafy vegetables and health hazards of human consumption remain rarely documented. Therefore, a comprehensive understanding of the levels of trace elements in leafy vegetables and their potential human health risks through the consumption of urban grown leafy vegetables is crucial for making informed decisions that affect vulnerable populations and minimize potential health risks.

The study aims to evaluate toxic metal levels in *Brassica pekinensis* L. from two urban farms in Kinshasa—Lutendele and Cecomaf and assess associated human health risks through EDI, THQ, HI, CRI, and TCRI. This study will provide mitigation strategies and agricultural policies for ensuring regional food safety and protecting human health from heavy metal pollution.

## 2 Materials and methods

### 2.1 Study sites and plant sampling

**Fig. 1:**
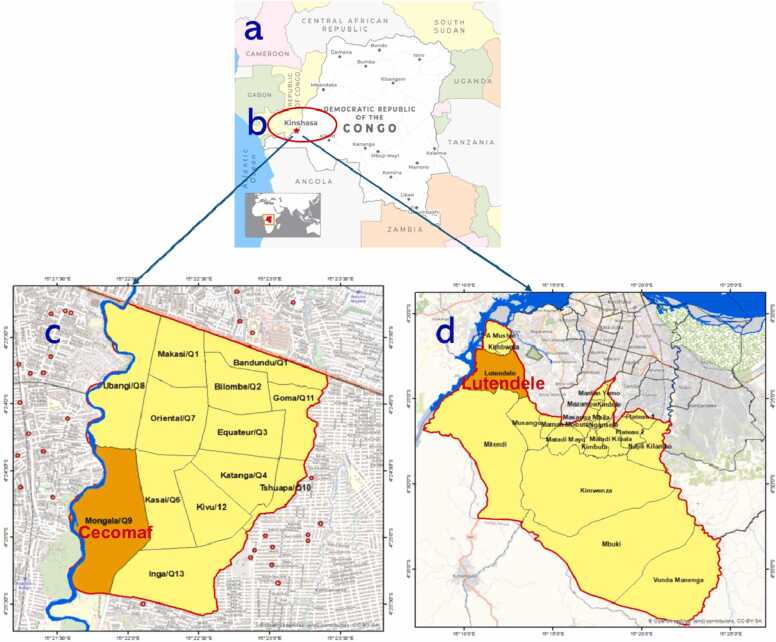
Sampling site adapted from the Google Earth indicating; (a) Africa continental map, (b) Map showing the location of Kinshasa City in the Democratic Republic of the Congo, (c) the Cecomaf and (d) the Lutendele station.

**Fig. 2:**
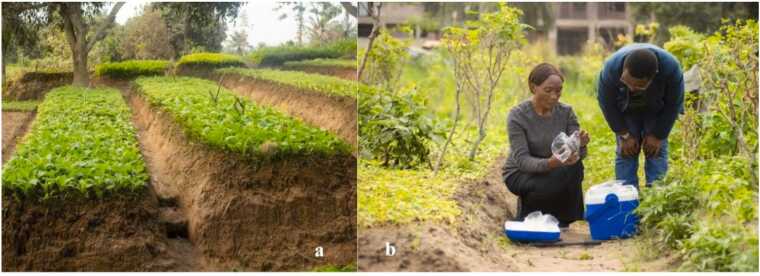
Photos of the largest site of Lutendele taken by Anaïs Kimpelo in August 2025, indicating; 2a: Field of *Brassica pekinensis Lour.* when the vegetable reached harvest stage, 2b: *Brassica pekinensis Lour.* samples treatment in field while sampling.

This research was performed in two main gardening areas of Kinshasa, capital city of DRC (Fig. 1); the station of Cecomaf is located in the commune of Ndjili and the station of Lutendele is located in the commune of Mont Ngafula. Both sites are characterized by the most important practices of intensive urban agriculture in Kinshasa. We selected these stations based on their cultivation areas and high vegetable production levels [48], [47]. The sampling sites are labelled: Cecomaf station (4 crop fields (n = 11); CS1A-CS1M, CS2A-CS2M-CS2P, CS3A-CS3M-CS3P, CS4A-CS4M-CS4P) and Lutendele station (4 crop fields (n = 8); labelled LS1A-LS1M, LS2A-LS2M, LS3A-LS3M, LS4A-LS4M). *Brassica pekinensis* L. leaf samples were collected in August 2025, when the vegetable was ready for harvest (Fig. 2: 2a)*.* For each sampling point (for good representability), about 500 g of *Brassica pekinensis* L. leaves were collected and homogenized. In each crop field, leaves were collected using the classical four quadrat sampling approach [13], [33], [47]. After field collection, samples were washed with deionized water to remove surface contamination, then stored in zip-lock polythene bags at 4°C (Fig. 2: 2b) and transported to the University of Geneva's analytical labs for analysis. *Brassica pekinensis* was selected due to its high consumption by the local population and its tendancy to accumulate trace metals. Its leafy structure and fast growth make it a suitable bioindicator.

### 2.2 Trace elements analysis in plant samples

Plant samples (leaves) were digested according to protocols described previously by Larras et al. [37] and Evangelou et al. [22], with some modifications. Briefly, the samples were rinsed with deionized water, lyophilized for 12 h, and ground under liquid nitrogen. For acid digestion, approximately 0.5 g of plant powder was added to 8 mL of nitric acid (HNO_3_, 65 %, Suprapur®, Merck KGaA, Darmstadt Germany) and 2 mL of hydrogen peroxide (H_2_O_2,_ 30 %, Merck KGaA, Darmstadt Germany). The reaction mixture was incubated at 105 °C for 16 h. The digestate was then cooled and diluted 100-fold with 1 % HNO_3_ (Suprapur®) and analyzed by inductively coupled plasma mass spectrometry (ICP-MS). Method accuracy was assessed using certified reference material (CRM BCR-482, European Commission - JRC, Geel, Belgium), which was prepared and analyzed under the same conditions as the plant samples. The coefficient of variation for triplicate ICP-MS measurements were below 2 % and three procedural blanks were consistently less than 1 % of the sample signals. Measured concentrations of metals in BCR-482 fell within the certified range, with recovery rates above 93 %.

### 2.3 Mercury analysis plant samples

Total mercury (Hg) concentrations in plant samples were determined using an Atomic Absorption Spectrophotometer (Advanced Mercury Analyser AMA-254, Altecs.r.l., Czech Republic), following the method of Roos-Barraclough et al. [52]. This technique involves thermal decomposition of the sample, amalgamation of released mercury on a gold trap, and quantification by AAS. The detection limit (3 SD blank) was 0.005 mg kg^−1^, with a reproducibility of better than 5 %. Concentrations are expressed on a dry weight basis. Accuracy was validated using the same certified reference material (CRM BCR-482). The measured Hg concentrations (0,46 mg kg^−1^) were within the certified range (0,48 mg kg^−1^), with recovery rates around 96 %.

### 2.4 Health hazard evaluation for Brassica pekinensis L. consumption

The potential health risk associated with consumption of contaminated *Brassica pekinensis* L. was evaluated by comparing the metal values measured in *Brassica pekinensis* L. leaves with the threshold levels (FAO/WHO 2000; 2004; 2006) and the standards for trace elements in food [35]. This study also assessed potential health risks by calculating the indices (1) the estimated daily intake (EDI), (2) the target hazard quotient (THQ) and (3) the hazard index (HI) as described in previous studies [36], [47], [71] (USEPA 1989a; 2018).

#### 2.4.1 Estimated daily intake (EDI)

The Estimated Daily Intake (EDI) of each heavy metal from BPL consumption was calculated using the following Equation (Eq) (1).:

$${\mathrm{EDI}}_{i}=\frac{C_{i}\mathrm{xDIR}}{\mathrm{BW}}$$

where: EDI_i_ (μg/kg BW/day) is the estimated daily intake of heavy metal i, C_i_ (mg kg^−1^) is the concentration of heavy metal i in BPL leaves, DIR is the daily ingestion rate of BPL leaves (60 g/day for local inhabitants), BW is the average body weight (58 kg) [68], [69].

#### 2.4.2 Potential non-carcinogenic health risk (THQ)

To evaluate the potential non-carcinogenic health risk from consuming *Brassica pekinensis* L, the Target Hazard Quotient (THQ) was calculated following the methodologies established by United States Environmental Protection Agency (USEPA) (1989b) as indicated in Eq (2):

$${\mathrm{THQ}}_{i}=\frac{{\mathrm{EDI}}_{i}\mathrm{xEFxED}}{{\mathrm{RfD}}_{i}\mathrm{xAT}}x10^{-3}$$

where: RfD_i_ (mg/kg bw/day) is the oral reference dose for metal i (USEPA, 2015), EF is the exposure frequency (365 days/year), ED is the exposure duration (70 years), AT is the averaging time (25,550 days = 70 years × 365 days/year). The RfD values for the analyzed metals are: Arsenic (As): 3.0 × 10⁻⁴ mg/kg bw/day, Cadmium (Cd): 1.0 × 10⁻³ mg/kg bw/day, Copper (Cu): 4.0 × 10⁻² mg/kg bw/day, Iron (Fe): 7.0 × 10⁻¹ mg/kg bw/day, Manganese (Mn): 1.4 × 10⁻¹ mg/kg bw/day, Lead (Pb): 4.0 × 10⁻³ mg/kg bw/day, Zinc (Zn): 3.0 × 10⁻¹ mg/kg bw/day [70], [65].

#### 2.4.3 Hazard index (HI)

To assess the overall non-carcinogenic risk from multiple metals, the Hazard Index (HI) via consumption of *Brassica pekinensis* L was calculated using Eq (3):

$$\mathrm{HI}=\sum_{i=1}^{n}{\mathrm{THQ}}_{i}$$

An HI > 1 indicates adverse health effects due to combined exposure are likely to occur [8], whereas if HI < 1, the human health risk is possibly low or negligible

### 2.5 Statistical analysis

All analyses were performed in triplicate. The statistical analysis was carried out using XLSTAT software from Addinsoft [2] (Statistical and data analysis solution, New York, United States, https://www.xlstat.com). Spearman’s rank correlation coefficient was used to assess potential positive and negative relationships between the variables.

The Monte Carlo simulation was performed to estimate the variability and uncertainty of dietary exposure to different metals. Key parameters, including metal concentrations in foods, local consumption habits, age, and sex of the population, were modeled using appropriate probability distributions for each variable. A total of 10,000 iterations were conducted to generate distributions of Estimated Daily Intake (EDI) and to assess the probabilities of exceeding FAO/WHO thresholds. Analyses were carried out using R (version 4.3.1) with packages dedicated to probabilistic simulations, allowing the generation of histograms, summary statistics, and probabilities of exceeding health-based limits.

## 3 Results and discussion

### 3.1 Metal concentrations in Brassica pekinensis Lour

The levels of trace elements—including Ti, V, Cr, Mn, Fe, Co, Ni, Cu, Zn, As, Se, Mo, Ag, Cd, Sn, Sb, Ba, Pb, and Hg—in *Brassica pekinensis* L. are presented in Table 1. Among all trace elements observed, Cu (115.39 mg/kg), Cd (2.95 mg/kg) and Pb (30.94 mg/kg) were found to be the highest concentrations in *Brassica pekinensis* L. The Lutendele site exhibited higher concentrations than the Cecomaf site for Ti (39.77 mg kg⁻¹), V (4.38 mg kg⁻¹), Mn (968.05 mg kg⁻¹), Fe (2696.99 mg kg⁻¹), Co (0.89 mg kg⁻¹), As (0.39 mg kg⁻¹), Se (0.58 mg kg⁻¹), and Mo (3.55 mg kg⁻¹). Conversely, higher concentrations were recorded at the Cecomaf site for Cr (4.55 mg kg⁻¹), Ni (6.18 mg kg⁻¹), Cu (115.39 mg kg⁻¹), Zn (844.94 mg kg⁻¹), Ag (0.04 mg kg⁻¹), Cd (2.95 mg kg⁻¹), Sn (1.93 mg kg⁻¹), Sb (0.26 mg kg⁻¹), Ba (137.91 mg kg⁻¹), Pb (30.94 mg kg⁻¹), and Hg (0.058 mg kg⁻¹). The average concentrations of Cr, Cu, Cd, Pb, and Hg in samples from both sites exceeded the maximum permissible limits for human consumption as set by the Food and Agriculture Organization/World Health Organization (FAO/WHO, 2003). Samples from Lutendele had levels 3.5 times higher for Cr, 2.9 times for Cu, 29.5 times for Cd, 103.3 times for Pb, and 58 times for Hg compared to the recommended limits. Thus, heavy metal levels exceeded the limit values for *Brassica pekinensis* L, *suggesting* these trace elements may pose a threat to human health and raise food safety concerns. The highest Cd concentration observed in this study was consistent with Cd contamination of vegetables cultivated on a peri-urban farm in Vietnam [50]. Moreover, the Cd level observed in this study is much higher than that found in leafy vegetables in Northeast Thailand as reported by Tongprung et al. [63]. In addition, previous studies found that leafy vegetables accumulate greater levels of Cd than tubers [62]. Hamilton et al. [29] reported that the high accumulation of Cd in leafy vegetable could also be attributed to high bioavailability in the soil [29]. In particular, Pb, Cd, and Hg are the most occurring contaminants that surpass the safe limit of trace elements in *Brassica pekinensis Lour*, indicating that urban farming practices are poor, and that substantial improvement is needed in agricultural practices to mitigate human health risks. Therefore, at all sites, *Brassica pekinensis Lour* was not safe for human consumption based on Pb, Cd, and Hg levels.

**Table 1:** The mean of metal concentrations in mg kg^−1^ in *Brassica pekinensis Lour.*

Sample Name		Ti	V	Cr	Mn	Fe	Co	Ni	Cu	Zn	As	Se	Mo	Ag	Cd	Sn	Sb	Ba	Pb	Hg
Cecomaf																				
LOD		0.098	0.024	0.000	0.150	0.681	0.025	0.000	1.516	2.033	0.049				0.063				1.091	0.005
CS1A		4.34	0.75	1.16	171.20	519.24	0.24	2.30	**85.42**	844.94	0.10	0.00	0.66	0.04	**2.24**	0.92	0.16	107.85	**6.67**	**0.030**
CS1M		5.32	0.68	**1.31**	310.08	483.03	0.14	2.15	**56.78**	74.54	0.07	0.00	0.27	0.01	**0.70**	1.93	0.18	30.46	**11.34**	**0.027**
CS2A		3.10	0.43	0.59	1154.11	268.31	0.34	1.65	**77.74**	522.84	0.05	0.15	0.17	0.01	**1.29**	0.92	0.10	29.55	**3.97**	**0.050**
CS2M		4.58	1.14	**1.35**	230.86	603.46	0.17	1.74	**68.84**	74.18	0.12	0.13	0.59	0.01	**0.25**	0.60	0.12	14.67	**11.51**	**0.033**
CS2P		6.21	0.58	**1.35**	194.61	527.99	0.61	6.18	**115.39**	814.78	0.07	0.07	1.90	0.04	**2.95**	1.09	0.18	137.91	**7.29**	**0.054**
CS3A		11.66	0.72	1.08	129.60	572.69	0.68	3.55	32.05	615.81	0.08	0.13	2.58	0.03	**2.15**	1.12	0.12	92.58	**5.84**	**0.051**
CS3M		3.84	0.53	0.92	68.75	435.42	0.11	2.31	27.29	81.34	0.06	0.09	1.43	0.00	**0.38**	1.02	0.20	14.83	**5.93**	**0.023**
CS3P		34.50	3.12	**3.73**	124.83	1513.08	0.35	5.16	23.39	289.07	0.26	0.19	2.43	0.03	**0.78**	1.08	0.26	42.05	**30.94**	**0.042**
CS4A		12.06	0.63	1.12	65.34	594.98	0.19	2.37	22.51	257.88	0.07	0.11	1.41	0.01	**0.75**	0.83	0.15	44.29	**4.31**	**0.058**
CS4M		5.05	0.35	0.69	126.38	273.16	0.06	1.41	23.59	49.86	0.04	0.00	2.72	0.00	**0.11**	0.89	0.10	5.18	**2.39**	**0.013**
CS4P		36.59	4.27	**4.55**	377.68	1924.69	0.44	2.87	**93.92**	96.93	0.31	0.19	0.95	0.02	**0.26**	0.76	0.17	25.40	**24.69**	**0.044**
Lutendele																				
LS1A		39.77	3.16	**3.04**	338.41	1785.77	0.39	3.02	5.35	254.15	0.24	0.30	2.72	0.01	**0.22**	1.37	0.06	31.94	**2.78**	**0.013**
LS1M		28.95	1.85	**2.11**	293.70	1068.39	0.17	3.02	7.55	59.91	0.16	0.27	1.05	0.00	**0.11**	1.06	0.08	9.54	**1.89**	**0.009**
LS2A		6.35	1.06	**1.47**	289.62	741.50	0.18	3.02	7.87	153.28	0.12	0.21	1.99	0.01	**0.14**	1.02	0.06	11.87	**1.22**	**0.011**
LS2M		11.39	1.15	1.13	329.97	814.13	0.20	3.63	6.88	84.01	0.11	0.13	0.80	0.00	**0.18**	0.89	0.06	35.65	**1.42**	**0.005**
LS3A		35.82	4.38	**3.61**	488.09	2696.99	0.54	2.89	10.05	176.56	0.39	0.58	0.74	0.01	**0.47**	0.44	0.06	40.86	**3.91**	**0.023**
LS3M		18.62	2.28	**2.20**	236.75	1431.44	0.20	5.73	33.59	63.00	0.21	0.20	3.55	0.01	**0.08**	0.68	0.10	12.45	**3.23**	**0.007**
LS4A		8.40	1.98	**1.89**	968.05	1235.82	0.89	2.26	7.65	277.45	0.21	0.26	0.69	0.01	**0.57**	0.82	0.11	51.12	**2.73**	**0.019**
LS4M		15.16	1.77	**1.65**	361.68	1153.31	0.19	1.84	7.63	31.77	0.18	0.15	0.42	0.00	**0.17**	0.78	0.06	16.85	**2.40**	**0.028**
**WHO/FAO (2003).**				1.3					40						0.1				0.3	0.001
BCR 482	Reference value			4.12				2.47	7.03	100.6	0.85				0.56				40.9	0.48
	Detected value	26.24	4.31	4.11	47.73	1226.88	0.48	2.61	7.02	99.69	0.79	0.15	0.63	0.08	0.52	2.40	0.44	9.54	38.97	0.46

All analyses from sampling points were performed in triplicate and the standard deviation was less than 3 % of average.

Compared to previous studies, this research reports higher Cu concentrations in Brassica pekinensis L. than those found in *Amaranthus viridis* grown at the Cecomaf site [47]. However, Ngweme et al. [47] observed higher levels of Cr (9.12 mg kg⁻¹), Pb (30.94 mg kg⁻¹), and Cd (2.95 mg kg⁻¹) [47]. In contrast, studies from Cameroon, Ghana, and South Africa reported lower concentrations of Cu, Cr, Pb, and Hg in leafy vegetables compared to this study (Table 2). Notably, the Pb levels in this study were significantly higher than those reported in leafy vegetables cultivated in South India [45]. Additionally, Cu, Pb, and Cr concentrations were higher than those found in leafy vegetables from West Shewa, Ethiopia [23]. The elevated concentrations of trace elements in *Brassica pekinensis* Lour*’s* leaves are possibly due to the application of agrochemicals, including pesticides and fungicides commonly used in Congolese agriculture. These products often contain metals such as Pb, Zn, Cr, As, Ni, and Hg. Another possible factor is the use of phosphate fertilizers, which can carry trace metals [32], [47]. Additionally, since *Brassica pekinensis* L. is primarily grown in peri-urban areas—often near rivers or along busy roads—atmospheric deposition could also contribute to contamination [44]. For instance, a study evaluating the effects of road traffic on accumulating trace elements in vegetables found elevated levels of trace elements in urban gardens cultivated vegetables [7]. Based on these findings, regular follow-up and continuous monitoring of trace elements in urban farming of leafy vegetables in Kinshasa and assessing risk are strongly recommended.

**Table 2:** Concentration ranges of some trace elements in edible vegetables cultivated under similar climatic conditions.

Country	Plant	Cu	Cr	Pb	Cd	Reference
Congo DR	*Brassica pekinensis Lour.*	5.35–115.39	0.59–4.55	1.22–30.94	0.11–2.95	This study
Congo DR	*Amaranthus viridis*	0.34–33.89	0.27–9.12	0.01–127.05	0.01–2.98	Ngweme [47]
Cameroun	Lactuca sativa	18.35–27.45	3.326–3.94	0.39–3.76	0.98–1.43	Mewouo [43]
Ghana	Manihot esculenta	2.1	0.05	0.18	0.007	Bortey-Sam [14]
South Africa	Tomato	4.48–9.60	ND	ND	0.04–0.50	Bvenura, Afolayan [16]

ND: No determined

### 3.2 Assessment of potential non-carcinogenic health risks

The non-carcinogenic health risk parameters, including Estimated Daily Intake (EDI), Target Hazard Quotient (THQ), and Hazard Index (HI) for trace elements in *Brassica pekinensis* L., are presented in Table 3. The EDI values for manganese (Mn), iron (Fe), copper (Cu), zinc (Zn), arsenic (As), cadmium (Cd), and lead (Pb) were calculated based on consumption data for *Brassica pekinensis* L. Previous studies demonstrated that toxicological risks from heavy metal-contaminated food depend on both metal concentrations in the food and the amount consumed by individuals [28], [41]. Compared to the provisional tolerable daily intake limits set by the Joint FAO/WHO Expert Committee on Food Additives [35], EDI values for Cd exceeded recommended limits in samples CS1A, CS2A, CS2P, and CS3A from the Cecomaf site. Pb concentrations exceeded permissible limits in nearly all Cecomaf samples and in one sample (LS3A) from the Lutendele site, suggesting potential health risks associated with increased and regular consumption of *Brassica pekinensis* L. from these areas. The results of EDI values observed in this study are in agreement with previous findings, which reported high EDI values for trace elements like Cd due to consumption of contaminated vegetables from wastewater-irrigated peri-urban areas in India [27]. The toxicological significance of these findings is considerable. Cadmium (Cd) is a non-essential and highly toxic metal that tends to accumulate in plants tissus and, through the food chain, in human organs such as the kidneys and liver [25]. Chronic exposure to Cd can cause renal dysfunction, bone demineralization (Itai-Itai disease), and carcinogenic effects [51]. Since chromium toxicity depends strongly on its chemical form, speciation into Cr(III) and Cr(VI) is essential: Cr(VI) is highly toxic and carcinogenic, while Cr(III) is an essential trace element (DesMarais and Costa, 2019). Moreover, domestic processing such as washing, soaking, and cooking can reduce Cr(VI) levels or convert part of it to the less toxic Cr(III) form [59]. Therefore, combining total Cr analysis with chromium speciation and consideration of food preparation effects allows a more accurate assessment of potential health risks associated with toxic Cr(VI) in vegetables. Similarly, lead (Pb) is a potent neurotoxin that affects the nervous system, hematopoietic function, and cognitive development, particularly in children. Its persistence in the environment and bioaccumulation potential make it one of the most concerning trace metals from a public health perspective [26].

**Table 3:** Estimated Daily Intake (μg/kg BW/day), Target Hazard Quotient (THQ) and Hazard index (HI) of Mn, Fe, Cu, Zn, As, Cd and Pb for adults based on consumption of *Brassica pekinensis Lour* in two sites.

**Sample**	**EDI**							**THQi**							**HI**
	Mn	Fe	Cu	Zn	As	Cd	Pb	Mn	Fe	Cu	Zn	As	Cd	Pb	
Cecomaf															
CS1A	177.1	537.14	88.36	874.1	0.1	2.3	6.89	1.26	0.77	2.209	2.914	0.33	2.3	1.72	11.53
CS1M	320.78	499.69	58.74	77.11	0.08	0.7	11.7	2.29	0.71	1.469	0.257	0.25	0.7	2.93	8.64
CS2A	1193.9	277.56	80.42	540.9	0.06	1.3	4.11	8.53	0.4	2.01	1.803	0.19	1.3	1.03	15.28
CS2M	238.82	624.26	71.21	76.74	0.12	0.3	11.9	1.71	0.89	1.78	0.256	0.41	0.3	2.98	8.28
CS2P	201.32	546.2	119.4	842.9	0.07	3	7.54	1.44	0.78	2.984	2.81	0.24	3	1.88	13.18
CS3A	134.07	592.44	33.15	637	0.08	2.2	6.05	0.96	0.85	0.829	2.123	0.28	2.2	1.51	8.77
CS3M	71.117	450.43	28.24	84.14	0.06	0.4	6.14	0.51	0.64	0.706	0.28	0.21	0.4	1.53	4.28
CS3P	129.13	1565.3	24.19	299	0.27	0.8	32	0.92	2.24	0.605	0.997	0.9	0.8	8	14.47
CS4A	67.594	615.5	23.29	266.8	0.07	0.8	4.45	0.48	0.88	0.582	0.889	0.23	0.8	1.11	4.96
CS4M	130.74	282.58	24.41	51.58	0.04	0.1	2.48	0.93	0.4	0.61	0.172	0.13	0.1	0.62	2.98
CS4P	390.7	1991.1	97.16	100.3	0.32	0.3	25.5	2.79	2.84	2.429	0.334	1.06	0.3	6.39	16.11
Lutendele															
LS1A	350.08	1847.4	5.535	262.9	0.25	0.2	2.88	2.5	2.64	0.138	0.876	0.84	0.2	0.72	7.94
LS1M	303.83	1105.2	7.815	61.97	0.17	0.1	1.95	2.17	1.58	0.195	0.207	0.57	0.1	0.49	5.33
LS2A	299.61	767.07	8.144	158.6	0.13	0.1	1.26	2.14	1.1	0.204	0.529	0.42	0.1	0.31	4.85
LS2M	341.35	842.21	7.115	86.91	0.11	0.2	1.47	2.44	1.2	0.178	0.29	0.38	0.2	0.37	5.04
LS3A	504.92	2790	10.39	182.7	0.4	0.5	4.04	3.61	3.99	0.26	0.609	1.34	0.5	1.01	11.29
LS3M	244.92	1480.8	34.75	65.17	0.22	0.1	3.34	1.75	2.12	0.869	0.217	0.72	0.1	0.84	6.6
LS4A	1001.4	1278.4	7.915	287	0.22	0.6	2.82	7.15	1.83	0.198	0.957	0.72	0.6	0.71	12.15
LS4M	374.16	1193.1	7.898	32.87	0.19	0.2	2.49	2.67	1.7	0.197	0.11	0.62	0.2	0.62	6.11
Limit value set by **FAO/WHO (2003)**	-	-	700	1000	2.14	1	3.6								

In contrast, EDI values for Mn, Fe, Cu, Zn, and As remained below safety thresholds, indicating minimal risk from these elements. THQ is commonly used to assess the non-carcinogenic risks associated with exposure to hazardous chemicals in food. A THQ value below 1 indicates that adverse health effects are unlikely to occur to the exposed population, whereas values exceeding 1 suggest potential health concerns [4] (FAO/WHO, 2013**).** The THQ values for Mn, Fe, Cu, Zn, As, Cd, and Pb in *Brassica pekinensis* L are presented in Table 3. The values were found to be above one for most trace elements at most sites, except As. Previous studies reported that a THQ value higher than 1 for Pb could result in organ failure, neurological complications and increased blood pressure [21]. Another study reported that the potential carcinogenic risk was associated with Pb-contaminated vegetables grown in landfills [31]. Consequently, the findings underscore the significant health risks, including neurotoxic effects, linked to consuming *Brassica pekinensis* L, exposed to trace elements. Therefore, the study recommends adopting good agricultural practices in urban gardening in Kinshasa to decrease heavy metal contamination in leafy vegetables and associated health risks to the consumers. The Hazard Index (HI) represents the cumulative health risk of trace elements, calculated as the sum of individual THQ values. According to previous studies, an HI value of one or less than one is the safety threshold, with values above one indicating potential adverse health effects likely to occur [20]. The THQ and HI values observed in this study are in same order than the values found in studies performed in similar environments (e.g. [47]). In this study, the HI values ranged between 2.98 and 16.11 at Cecomaf and 4.85 and 12.15 at Lutendele, suggesting potential health risks for consumers of *Brassica pekinensis* L. from these sites, which include organ damage and increased cancer risk. Thus, according to HI values, *Brassica pekinensis* L. consumption was not safe.

**Fig. 3:**
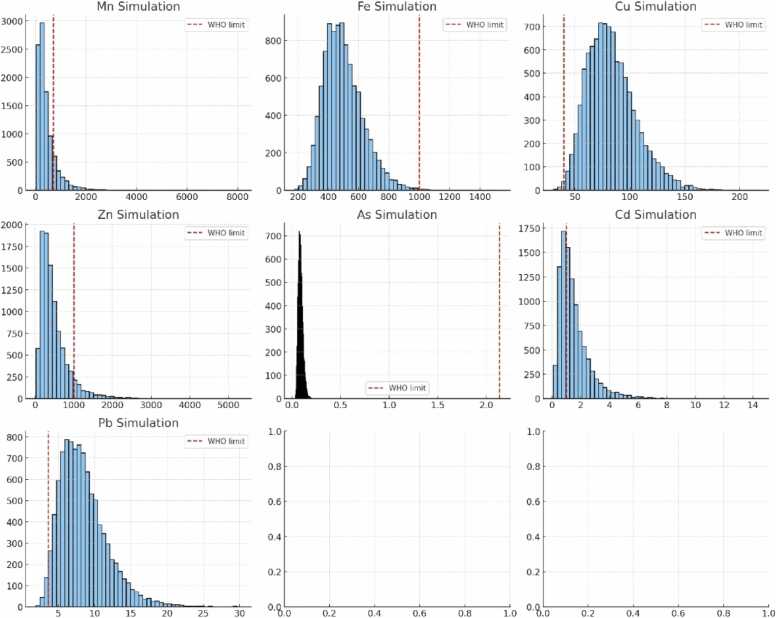
Histograms of simulated dietary metal exposures (Mn, Fe, Cu, Zn, As, Cd, Pb) compared to WHO limits.

The results of the Monte Carlo simulation (Fig. 3;Table 4) highlight significant variations in dietary metal exposure in the studied population, accounting for individual variability related to age, sex, and local consumption habits. Manganese (Mn) shows an asymmetric distribution, with a minority (∼16 %) of the population exceeding the WHO daily permissible limit. The risk is particularly notable among children and pregnant women, who are more sensitive to neurotoxic effects. Iron (Fe) shows largely safe exposure, with all simulated values below the WHO limit. The low dispersion suggests negligible risk for the general population, regardless of age or sex. Copper (Cu) is concerning, as nearly 99.6 % of individuals exceed the WHO limit. This high proportion indicates a very high risk of non-carcinogenic toxicity, especially for intensive local consumers. Zinc (Zn) presents a moderate risk: although most EDI values are below the limit, about 8 % of simulations exceed the WHO threshold. Children and regular consumers are the most exposed. Arsenic (As) remains generally safe, with exposures well below the WHO limit and no significant fraction exceeding the threshold. Cadmium (Cd) poses a high risk, with ∼62 % of simulations exceeding the WHO limit. The long tail of the distribution toward high values highlights the vulnerability of high-consumption or sensitive groups. Lead (Pb) represents the most critical risk, with nearly 98 % of values surpassing the WHO limit, indicating a major public health concern for the entire population. Cu, Pb, and Cd are the primary metals of concern in this population, necessitating monitoring and exposure reduction strategies. Mn and Zn present moderate risks, while Fe and As remain safe. The probabilistic approach enhances the reliability of risk assessment and the relevance of health recommendations.

**Table 4:** Probability of exceeding WHO limits for dietary metal intake estimated by Monte Carlo simulation.

Metal	WHO limit	Probability of exceedance
Mn	700	15.8 %
Fe	1000	0.25 %
Cu	40	99.6
Zn	1000	8.2 %
As	2.14	0 %
Cd	1	62 %
Pb	3.6	98.1 %

However, it is important to note that only one leafy vegetable was considered in this study to estimate possible health risks from the study area. Therefore, the authors ascertain that continuous assessment of heavy metal levels and associated health risks on consumption might be underestimated in other vegetables cultivated in this region, which is vital for protecting consumers from health risks and implementing mitigation strategies.

### 3.3 Spearman correlation between parameters

The Spearman correlation coefficients calculated between the trace elements analyzed from *Brassica pekinensis* L. at the Cecomaf and Lutendele sites are presented in Table 5. At the Cecomaf site, several statistically significant positive correlations (p < 0.05) were observed, notably between Cr and As (R² = 0.855), Cr and Pb (R² = 0.929), and Cd and Hg (R² = 0.564). These strong correlations suggest that these metal pairs may originate from common sources or share similar transport pathways and accumulation [10]. A similar trend was observed at the Lutendele site, where significant positive correlations were also recorded—specifically between Cr and As (R² = 0.910), Cr and Pb (R² = 0.881), and Cd and Hg (R² = 0.524)—leading to the same inference regarding potential common sources or pathways. The results showed no significant negative correlations at both sites. The higher concentrations observed in the vegetable sample may stem from interactions between trace elements [6].

**Table 5:** Spearman correlation coefficients between heavy metal concentrations in Cecomaf and Lutendele sites.

Cecomaf	Cr	Mn	Fe	Co	Ni	Cu	Zn	As	Se	Ag	Cd	Sn	Sb	Ba	Pb	Hg
Ti	**0.661**	−0.173	**0.791**	0.491	**0.673**	−0.164	0.045	**0.587**	0.401	0.370	−0.009	0.018	0.325	0.264	0.464	0.445
Cr		0.187	**0.843**	0.392	**0.565**	0.310	0.055	**0.855**	0.335	**0.531**	0.005	−0.087	**0.569**	0.205	**0.929**	0.187
Mn			−0.073	0.255	−0.264	**0.773**	0.045	0.147	0.175	0.169	0.036	−0.050	−0.256	−0.091	0.227	0.045
Fe				0.482	**0.609**	0.009	0.064	**0.899**	**0.576**	0.445	−0.055	−0.282	0.334	0.173	**0.727**	0.409
Co					**0.736**	0.427	**0.745**	**0.505**	**0.535**	**0.811**	**0.673**	0.251	0.101	**0.664**	0.291	**0.755**
Ni						0.091	**0.555**	**0.505**	0.341	**0.675**	**0.536**	0.378	**0.632**	**0.664**	0.473	**0.582**
Cu							0.391	0.229	−0.018	0.497	0.336	−0.014	−0.018	0.245	0.273	0.145
Zn								0.202	0.097	**0.829**	**0.955**	0.360	0.133	**0.882**	0.000	**0.582**
As									**0.516**	**0.591**	0.064	−0.184	0.365	0.220	**0.835**	0.220
Se										0.135	−0.041	−0.268	0.046	−0.111	0.378	0.456
Ag											**0.783**	0.301	0.274	**0.834**	0.431	**0.501**
Cd												**0.556**	0.183	**0.927**	−0.027	**0.564**
Sn													0.429	**0.515**	0.036	0.009
Sb														0.284	**0.673**	−0.082
Ba															0.091	**0.618**
Pb																−0.009
Lutendele	Cr	Mn	Fe	Co	Ni	Cu	Zn	As	Se	Ag	Cd	Sn	Sb	Ba	Pb	Hg
Ti	**0.833**	0.095	**0.738**	0.168	0.073	−0.214	0.000	**0.659**	**0.619**	0.056	0.048	0.048	−0.164	0.000	**0.667**	0.143
Cr		0.190	**0.905**	0.419	−0.049	0.238	0.286	**0.910**	**0.810**	**0.507**	0.167	−0.214	0.136	0.143	**0.881**	0.310
Mn			0.429	**0.683**	−0.805	−0.167	0.476	0.467	0.333	0.056	**0.905**	−0.238	−0.027	**0.810**	0.333	**0.738**
Fe				**0.695**	−0.171	0.214	0.381	**0.970**	**0.619**	**0.507**	0.405	−0.405	0.109	0.476	**0.976**	0.429
Co					−0.233	0.120	**0.778**	**0.681**	0.335	**0.567**	**0.814**	−0.371	0.192	**0.922**	**0.659**	0.323
Ni						0.024	−0.122	−0.295	−0.244	0.058	−0.561	0.195	0.084	−0.366	−0.098	−0.927
Cu							−0.024	0.275	0.048	**0.507**	−0.214	−0.762	0.327	−0.024	0.381	0.119
Zn								0.467	**0.524**	**0.732**	**0.762**	0.119	0.109	**0.667**	0.286	0.167
As									**0.719**	**0.624**	0.443	−0.359	0.123	0.443	**0.934**	**0.551**
Se										**0.507**	0.381	0.119	0.027	0.143	**0.524**	0.381
Ag											0.282	−0.169	0.194	0.282	**0.507**	0.169
Cd												−0.095	−0.109	**0.905**	0.286	**0.524**
Sn													−0.136	−0.381	−0.548	−0.310
Sb														0.027	0.218	−0.191
Ba															0.429	0.357
Pb																0.357

The pairs of variables from both tables with positive correlation and p < 0.05 tend to increase together. For the pairs with negative correlation coefficients and p < 0.05, one variable tends to decrease while the other increases. For pairs with P > 0.05, there is no significant relationship between the variables. Statistically significant coefficients (p < 0.05) are in bold.

## 4 Conclusion

This study indicates significant contamination of *Brassica pekinensis* L*.* by trace elements at both the Cecomaf and Lutendele sites in Kinshasa, Democratic Republic of the Congo. Concentrations of Cr, Cu, Cd, Pb, and Hg in plant tissues exceeded the maximum permissible limits established by FAO/WHO guidelines, with Cecomaf generally showing higher levels of Cu, Cd, Pb, and Hg, while Lutendele exhibited higher levels of Mn, Fe, As, and Mo. *Brassica pekinensis* L was therefore not safe for human consumption due to its elevated toxic metal concentration. It is likely that high metal concentrations are linked to intensive use of agrochemicals, phosphate fertilizers, and air pollution from peri-urban agriculture near polluted rivers and road vehicle emissions. According to the health risk assessment, the consumption of *Brassica pekinensis* L from these sites poses significant health risks. For most metals except As, EDI values exceeded safe limits, while THQ values exceeded 1, suggesting non-negligible non-carcinogenic health risks. In addition, all sampling sites showed Hazard Indexes (HI) well above the safety threshold, indicating the likelihood of cumulative toxic effects over time. The results of the Monte Carlo simulation reinforced these findings by accounting for variability and uncertainty in dietary exposure. The probabilistic approach showed that Pb, Cu, and Cd were the primary metals of concern, with a high proportion of simulated EDI values exceeding WHO limits, particularly among children and regular consumers. Mn and Zn posed moderate risks, while Fe and As remained largely within safe limits. This analysis underscored the importance of considering individual differences in age, sex, and local consumption habits, providing a more comprehensive and reliable assessment of health risks.

These outcomes underscore the urgent need for monitoring heavy metal levels in vegetables consumed by local populations, especially those cultivated in urban and peri-urban zones. Spearman correlation analysis revealed strong positive relationships among specific heavy metal pairs—particularly Cr/As, Cr/Pb, and Cd/Hg—suggesting common anthropogenic sources or similar environmental transport mechanisms. These findings emphasize the role of localized pollution sources in shaping contamination patterns. Considering these results, there is a critical need for implementing strict environmental and agricultural regulations, promoting safer farming practices, and raising public awareness about the potential health risks associated with consuming contaminated vegetables. This study strongly recommends policymakers, stakeholders, and municipal water resources management authorities to work together to develop guidelines, safety policies, and effective implementation to mitigate pollution sources. The study will make farmers and the public aware of the importance of food safety and good agricultural practices. In addition, the authors recommend adopting sustainable, eco-friendly approaches to urban agriculture to reduce the use of chemical fertilizers and pesticides to prevent heavy metal accumulation.

## CRediT authorship contribution statement

**Poté John:** Writing – original draft, Visualization, Validation, Software, Project administration, Methodology, Investigation, Funding acquisition, Conceptualization. **Crispin Mulaji:** Writing – original draft, Visualization, Validation, Software, Resources, Methodology, Investigation, Conceptualization. **Periyasamy Sivalingam:** Writing – original draft, Visualization, Validation, Software, Methodology, Investigation, Conceptualization. **Anaïs M. Kipelo:** Writing – original draft, Visualization, Validation, Investigation, Formal analysis, Conceptualization. **Georgette N. Ngweme:** Writing – original draft, Visualization, Validation, Methodology, Conceptualization. **Emmanuel K. Atibu:** Writing – original draft, Visualization, Validation, Methodology, Formal analysis, Data curation, Conceptualization. **Masi Jothilakshmi:** Writing – original draft, Visualization, Validation, Software, Formal analysis. **Al Salah Dhafer M. M.:** Writing – original draft, Visualization, Validation, Methodology, Data curation, Conceptualization.

## Compliance with Ethical Standards

We confirm that field studies did not involve endangered and protected species. The funders had no role in study design, data collection and analysis, decision to publish, or preparation of the manuscript.

## Funding

This research was funded by Leading House for The Middle East and North Africa “Consolidation Grants, MENA, 2023” and King Abdulaziz City for Science and Technology, and the Saudi Arabian Cultural Mission (Scholarship no. 11432), and supported by department F.-A. Forel within triplicate collaboration between University of Geneva, University of Kinshasa (DRC) and Pedagogic National University (DRC) through PRCERSE (Programme de Renforcement des Capacités de l’Enseignement et de la Recherche en Sciences de l’Environnement).

## Declaration of Competing Interest

The authors declare that they have no known competing financial interests or personal relationships that could have appeared to influence the work reported in this paper.

## Data Availability

No data was used for the research described in the article.
